# RETRACTED: El-Far et al. Nanonutraceuticals: Anti-Cancer Activity and Improved Safety of Chemotherapy by Costunolide and Its Nanoformulation against Colon and Breast Cancer. *Biomedicines* 2021, *9*, 990

**DOI:** 10.3390/biomedicines12102305

**Published:** 2024-10-10

**Authors:** Ali H. El-Far, Kavitha Godugu, Taher A. Salaheldin, Noureldien H. E. Darwish, Amna A. Saddiq, Shaker A. Mousa

**Affiliations:** 1 Department of Biochemistry, Faculty of Veterinary Medicine, Damanhour University, Damanhour 22511, Egypt; ali.elfar@damanhour.edu.eg; 2 Pharmaceutical Research Institute, Albany College of Pharmacy and Health Sciences, Rensselaer, NY 12144, USA; kavitha.godugu@acphs.edu (K.G.); taher.salaheldin@acphs.edu (T.A.S.); noureldien.darwish@acphs.edu (N.H.E.D.); 3 Department of Biology, College of Sciences, University of Jeddah, Jeddah 21589, Saudi Arabia; aansaddiq@kau.edu.sa

The journal retracts the article, “Nanonutraceuticals: Anti-Cancer Activity and Improved Safety of Chemotherapy by Costunolide and Its Nanoformulation against Colon and Breast Cancer” [1], cited above.

Following publication, concerns were brought to the attention of the publisher regarding the reliability of the figures presented in this article [1].

Adhering to our complaint’s procedure, an investigation was conducted by the Editorial Office and Editorial Board, with input from the Albany College of Pharmacy and Health Sciences. During this process, the authors were unable to satisfactorily explain the similarities between Figure 6A,C nor provide raw material for the Editorial Board to review.

As a result, the Editorial Board has lost confidence in the reliability of the findings and decided to retract this publication [1] in agreement with the recommendations of the Albany College of Pharmacy and Health Sciences and as per MDPI’s retraction policy (https://www.mdpi.com/ethics#_bookmark30, accessed on 8 August 2024). 

This retraction was approved by the Editor-in-Chief of the journal *Biomedicines*. 

The authors did not agree to this retraction.

## References

[1] El-Far A.H., Godugu K., Salaheldin T.A., Darwish N.H.E., Saddiq A.A., Mousa S.A. (2021). RETRACTED: Nanonutraceuticals: Anti-Cancer Activity and Improved Safety of Chemotherapy by Costunolide and Its Nanoformulation against Colon and Breast Cancer. Biomedicines.

